# Recent Advances in Nutrition for Disease Prevention and Sports Performance Enhancement

**DOI:** 10.3390/nu15051170

**Published:** 2023-02-26

**Authors:** Pedro L. Valenzuela

**Affiliations:** 1 Research Institute of the Hospital 12 de Octubre (“Imas12”, PaHerg Group), 28041 Madrid, Spain; pedro.valenzuela92@gmail.com; Tel.: +34-91-779-2713; 2 Department of Systems Biology, University of Alcalá, 28871 Madrid, Spain

The important role of nutrition on both health and sports performance, and particularly its joint association with physical exercise, is becoming increasingly clear in recent years. The present Special Issue, entitled “Recent Advances in Nutrition for Disease Prevention and Sports Performance Enhancement” reflects the great progress that is being made in this field, including different studies that address important topics related to nutrition from an integrative and multi-domain perspective.

On the one hand, we find studies focused on the role of nutrition for disease prevention. Xu et al. find an association between the plasma levels of oxylipins (i.e., the oxidation products of polyunsaturated fatty acids such as omega-3 and omega-6) on the fecal microbiota composition of young adults [1], which can have important implications for metabolic health [2]. Using a mouse model subjected to castration to accelerate sarcopenia features (e.g., loss of muscle mass and function), Martins et al. provide preliminary evidence on the effects that some phytoanabolic extracts obtained from Eurasian plants can have, particularly in combination with resistance training, on different indicators such as body composition, physical function, skeletal muscle/adipose tissue histology, and other biochemical indicators (e.g., cytokines, blood cholesterol) [3]. Moreover, Bellini et al. assessed the effect of postprandial walking on glycemic responses in healthy adults, finding that a 30-min postprandial brisk walking session improved the glycemic response after meals with different carbohydrate content and macronutrient composition [4]. The findings by Bellini et al. might be of clinical relevance, particularly given the potential detrimental effect of postpandrial hyperglycemia for cardiometabolic health [5]. Finally, a review article by Morales et al. summarizes the role of not only nutrition but also other components of the so-called “exposome” (e.g., physical activity, body weight, smoking status, sleep habits) on immune health, which is a timely topic particularly if considering situations such as the recent COVID-19 pandemic [6].

On the other hand, in this Special Issue, we find other studies more related to advances in the context of sports. Martinez-Rodriguez et al. report the body composition and anthropometric characteristics of 36 male players of the Spanish National Beach Handball Team, a sport that has received little attention to date in scientific research [7]. Focusing on the field of sports supplements and using a meta-analytical approach (18 studies included), Gomez-Bruton et al. report that acute caffeine supplementation improves team sport performance in female athletes, as reflected by increases in different outcomes such as specific team-sport skills as well as on countermovement jump height or total body impacts [8]. Additionally, focused on the field of sports supplements, Su et al. explored the effects of different doses of resveratrol on performance during a downhill running test to exhaustion (which was designed to provoke high levels of exercise-induced muscle damage) as well as on different markers of inflammation and energy metabolism [9]. Of note, high-dose resveratrol increased the time to exhaustion during the downhill running test and reduced the levels of pro-inflammatory markers such as tumor necrosis factor-α. It also enhanced the mRNA expressions of sirtuin 1, glucose transporter 4, AMP-activated protein kinase α1, and AMP-activated protein kinase α2 [9]. This preliminary evidence suggests, therefore, that resveratrol supplementation (in this case, for 4 weeks) might enhance exercise performance and attenuate inflammation after exercise-induced muscle damage. In the same line, Tanabe et al. also review the role of different dietary supplements with potential anti-inflammatory or antioxidant effects (e.g., curcumin, tart cherry juice, beetroot juice, quercetin, and isothiocyanate) for attenuating exercise-induced muscle damage and the associated delayed-onset muscle soreness, highlighting the potential beneficial effect of some of them but also the need for well-controlled studies [10]. It seems, therefore, reasonable that many athletes consume supplements with the aim of improving their performance, as summarized in the scoping review by Daher et al., who also highlight the need for rigorous research in this field (e.g., trying to homogenize factors such as the definition of dietary supplements or the definition of use) [11]. This is indeed the topic of the study by Moreno et al., who analyzed a sample of 102 male and female competitive swimmers and concluded that 86.9% of them had consumed sports supplements, with caffeine, sports drinks/bars, and vitamin C being among the most widely consumed and with no differences between genders or performance level [12]. 

Great advances are being made in the field of nutrition, which can lead to improvements in both the general health of the population and the performance of athletes. More high-quality evidence is needed on all the topics mentioned above, but hopefully these and other studies will lead the way and open new fields of research. 
